# Chlamydiaphage φCPG1 Capsid Protein Vp1 Inhibits *Chlamydia trachomatis* Growth via the Mitogen-Activated Protein Kinase Pathway

**DOI:** 10.3390/v8040099

**Published:** 2016-04-14

**Authors:** Yuanli Guo, Rui Guo, Quan Zhou, Changgui Sun, Xinmei Zhang, Yuanjun Liu, Quanzhong Liu

**Affiliations:** Dermatology and Venereology Department, Tianjin Medical University General Hospital, No. 154 Anshan Road, Heping District, Tianjin 300052, China; lvninle@sina.com (Y.G.); 13902052977@163.com (R.G.); peter-pf.liu@cn.ey.com (Q.Z.); 2008021327@tmu.edu.cn (C.S.); hongheifeiying@sina.com (X.Z.); liuyuanjun1980@163.com (Y.L.)

**Keywords:** *Chlamydia trachomatis*, mitogen-activated protein kinase pathway, chlamydiaphage, capsid protein Vp1, azithromycin

## Abstract

*Chlamydia trachomatis* is the most common cause of curable bacterial sexually transmitted infections worldwide. Although the pathogen is well established, the pathogenic mechanisms remain unclear. Given the current challenges of antibiotic resistance and blocked processes of vaccine development, the use of a specific chlamydiaphage may be a new treatment solution. φCPG1 is a lytic phage specific for *Chlamydia caviae*, and shows over 90% nucleotide sequence identity with other chlamydiaphages. Vp1 is the major capsid protein of φCPG1. Purified Vp1 was previously confirmed to inhibit *Chlamydia trachomatis* growth. We here report the first attempt at exploring the relationship between Vp1-treated *C. trachomatis* and the protein and gene levels of the mitogen-activated/extracellular regulated protein kinase (MAPK/ERK) pathway by Western blotting and real-time PCR, respectively. Moreover, we evaluated the levels of pro-inflammatory cytokines interleukin (IL)-8 and IL-1 by enzyme-linked immunosorbent assay after Vp1 treatment. After 48 h of incubation, the p-ERK level of the Vp1-treated group decreased compared with that of the *Chlamydia* infection group. Accordingly, *ERK1* and *ERK2* mRNA expression levels of the Vp1-treated group also decreased compared with the *Chlamydia* infection group. IL-8 and IL-1 levels were also decreased after Vp1 treatment compared with the untreated group. Our results demonstrate that the inhibition effect of the chlamydiaphage φCPG1 capsid protein Vp1 on *C. trachomatis* is associated with the MAPK pathway, and inhibits production of the pro-inflammatory cytokines IL-8 and IL-1. The bacteriophages may provide insight into a new signaling transduction mechanism to influence their hosts, in addition to bacteriolysis.

## 1. Introduction

*Chlamydia trachomatis* is the most common cause of curable bacterial sexually transmitted infection worldwide because of their prevalence and potentially devastating reproductive consequences, including pelvic inflammatory disease, infertility, and ectopic pregnancy. The number of chlamydial-infected citizens reached 1,422,976 in the United States in 2012 [1]. In China, the prevalence of chlamydial infection has also increased, and was reported to be 2.6 and 2.1 per 100 individuals for women and men, respectively [2]. Although the basic biology of the pathogen is well established, the pathogenic mechanisms are still unclear. Even worse, the condition of antibiotics resistance is becoming more and more serious. Therefore, further exploration of the *Chlamydia* infection mechanisms is sorely needed to develop new treatment methods to overcome the current public health challenges. Given the condition of a blocked process of developing a vaccine for *Chlamydia trachomatis*, use of a specific chlamydiaphage may be a new solution.

Chlamydiae have an obligate intracellular developmental cycle that alternates between the infectious elementary body and the replicative reticulate body. Six bacteriophages have been isolated from the Chlamydiae: Chp1, Chp2, Chp3, Chp4, φCPG1, and φCPAR39. All of the chlamydiaphages share similar features; they are small, icosahedral T = 1 particles containing circular, single-stranded DNA genomes, and molecular characterization revealed that they belong to the virus family Microviridae [3,4,5]. φCPG1 is a lytic phage specific to *Chlamydia caviae*, which is a natural parasite of the guinea pig. The genome of φCPG1 contains five open reading frames, which encode the capsid proteins Vp1, Vp2, and Vp3. Capsid proteins, especially Vp1, play essential roles in adhesion and invasion. Our research group has compared the Vp1 of all *Chlamydia* phages found until now and recovered nearly 98% comparability among them. Even though a specific bacteriophage of *C. trachomatis* has not yet been detected, the high homology among the known chlamydiaphages allows for the reasonable assumption of cross-reaction among species. The phage host range is specified by a peptidic loop of Vp1, and sequence variation within the loop is consistent with the observed overlapping host ranges of the phages [6]. Vp1 also plays an important role in the interaction between *Chlamydia* and its host cell. Our research group successfully expressed and purified Vp1, which was incubated with *C. trachomatis* and confirmed to inhibit its growth [7]. However, the specific mechanism underlying this inhibition effect has not yet been explored.

The mechanisms of *C. trachomatis* infection remain mysterious. Most research conducted in this field thus far has considered the inflammation-induced pathological damage [8,9]. The *C. trachomatis**-***induced inflammation reaction is mediated by signaling pathways in the host cell that are triggered after infection, including the mitogen-activated protein kinase (MAPK) pathway, Janus-activated kinase-signal transducer and activator of transcription (JAK/STAT) pathway, and nuclear factor-kappa B (NF-κB) pathway, among others. In particular, the MAPK pathway plays a major role in post-infection signal transduction, and the growth of *C. trachomatis* depends on the MAPK/ERK (mitogen-activated protein kinase / extracellular regulated protein kinases) pathway. During the course of incubation in the host, *C. trachomatis* enters into its own growth cycle, which induces increased phosphorylation levels of ERK protein in the host cell. Once the growth cycle is blocked with U0126, a specific inhibitor of MEK, the infection rate decreases, the major outer membrane protein (MOMP) level of *C. trachomatis* is reduced, and the phosphorylation level of ERK1/2 of the host is downregulated [10]. These facts reveal that *C. trachomatis* modulates the host MEK/ERK pathway and relies on it for its growth. In response to the increase in p-ERK, the host cell will release pro-inflammatory factors such as interleukin (IL)-1, IL-8, and tumor necrosis factor (TNF)-α [11]. IL-1 and IL-8 are considered the most significant factors contributing to host cell inflammatory injury in *C. trachomatis* infection [12,13].

Azithromycin has been the recommended antibiotic for treatment of *C. trachomatis* infection according to the guideline. Previous studies reported that azithromycin inhibits cytokine production, which may contribute to its therapeutic effect in the treatment of chronic *C. trachomatis* infection [14]. In addition, phosphorylation of ERK was found to be inhibited after azithromycin treatment in epithelial cells obtained from women with recurrent infection [15]. Traditionally, bacteriolysis is considered the main mechanism by which a bacteriophage inhibits the growth of its host. However, there may be alternative mechanisms that have not yet been explored, which could provide useful insights for the development of new treatments. In the present study, we aimed to address the following remaining questions related to the mechanism of *Chlamydia* growth inhibition by Vp1. In particular, we investigated whether Vp1 might exert its inhibition effect via the MAPK pathway similar to azithromycin, and compared the inhibition effect of Vp1 and azithromycin.

## 2. Materials and Methods

### 2.1. McCoy Cell Culture

McCoy cells (mouse fibroblast L cell origin) were purchased from the Chinese Academy of Medical Sciences, and stored in the Tianjin Institute of Sexually Transmitted Diseases, China. They were grown in minimal essential medium supplemented with 10% fetal bovine serum for *Chlamydia* culture. When the cells were distributed compactly and uniformly as a single layer, they were transferred to 6-well plates for continuous incubation and *Chlamydia* infection.

### 2.2. C. trachomatis Infection

The monolayer McCoy cells were pretreated with 30 μg/mL of DEAE-D for 30 min to increase the susceptibility of infection. The *C. trachomatis* strain used in this study was the E serotype strain that is maintained in our laboratory. The strain was pretreated with two freeze-thawing cycles, oscillation, and centrifugation at 500× g for 5 min. The plates were further centrifuged at 500× g at 32 °C for 1 h to facilitate *Chlamydia* adhesion. After 2 h, the wells of the plate were overlaid with culture medium containing 1 mg/L cycloheximide, and incubated for 44–48 h. The majority of cells were collected in transport culture medium, and the remaining cells were fixed with methanol and stained with iodine dye to observe the residual inclusions to verify that the infective rate was over 90%.

### 2.3. Vp1 Expression, Identification, and Purification

The stored *E. coli* bacteria that contained Vp1-pET30a(+) [16] were incubated in kanamycin-resistant Luria-Bertani medium for 12–16 h. The bacteria were amplified in the shaker and Vp1 expression was induced with 0.03 mM isopropyl-β-d-thiogalactopyranoside (IPTG) at 30 °C for 3 h until reaching the logarithmic phase (OD 0.6–0.8 in 600 nm). The bacterial solution was centrifuged and the sediment was treated with 4 mg/mL lysozyme and 3% Triton-X100. The suspension was ultrasonicated and centrifuged. The sediment was blended in PBS with 6 mol/L urea and identified by sodium dodecyl sulfate-polyacrylamide gel electrophoresis (SDS-PAGE). The purification of Vp1 was achieved with the PAGE recovery method, renaturated by dialysis and quantified for further use. LPS (Lipopolysaccharides) was neutralized less than 0.1 EU/mL by ToxinEraser^TM^ endotoxin removal resin kit from GenScript (Piscataway, NJ, USA). Vp1 was confirmed without toxic effect on McCoy cells by MTT (3-(4,5-dimethyl-2-thiazolyl)-2,5-diphenyl-2-H-tetrazolium bromide) method.

### 2.4. Treatments and Experimental Design

The McCoy cells were incubated with or without *C. trachomatis*, Vp1 and azithromycin, and classified into 6 experimental groups (Table 1). *C. trachomatis* was infected at 3 × 10^5^ IFU/mL after purification and quantification. The inoculation loads of Vp1 and azithromycin were 60 μg/mL and 25 μg/mL, according to previous reports^12^ and the results of preliminary experiments. After 48 h, the inclusions were counted under the microscope and stained with iodine dye.

### 2.5. Western Blotting

The cells were incubated at 0, 12 h, 24 h, 36 h, or 48 h, according to treatment, and the cellular proteins was extracted by the RIPA (Radio Immunoprecipitation Assay) method. The total proteins were separated by SDS-PAGE. The polyvinylidene fluoride (PVDF) membrane (Millipore Immobilon-P, Darmstadt, Germany). The monoclonal antibodies of p-ERK1/2 and total ERK1/2 (Cell Signaling Technology, Danvers, MA, USA) were added to the relevant treatment groups at a dilution of 1:1000. The corresponding secondary antibodies (Cell Signaling Technology) were added at dilutions of 1:5000–1:10000. The PVDF membrane was exposed and photographed after development, and the grey value ratio was used for evaluation determined with Gel-Pro Analyzer Software (Media Cybernetics, Rockville, MD, USA). The p-ERK1/2 level was standardized by the grey value ratio of ERK1/2. The experiment was repeated three times and the results are presented as the mean and standard deviation.

### 2.6. Real-Time PCR

The PCR protocols were performed according to the methods described by Wang and Seed [17]. In brief, cells in Groups M, C, C + V, and C + A were incubated for 48 h in 6-well plates, and the cells were dissociated and centrifuged at 500× g for collection according to the treatment groups. Total RNA was extracted by the TRNzol method and its purity and concentration were verified on a spectrophotometer. Reverse transcription was carried out with the PrimeScript RT reagent Kit (Qiagen, Hilden, Germany) with gDNA Eraser (Qiagen) for RT-PCR. The primers were synthesized by Invitrogen (Carlsbad, CA, USA) (Table 2). The PCR program was carried out at 95 °C 30 s, (95 °C 5 s, 60 °C 40 s) × 45 with SYBR® Premix Ex Taq™ II (Tli RNaseH Plus) (Takara Bio, Dalian, China), ROX plus (Takara Bio) on an ABI 7500 system (Applied Biosystems, Foster City, CA, USA). The results were quantitatively analyzed based on *Actin* by the 2^−ΔΔct^ method.

### 2.7. IL-1 and IL-8 Enzyme-Linked Immunosorbent Assay

The supernatants of treated cells from the different groups were collected at various time points of incubation (0, 12 h, 24 h, 36 h, 48 h) and subjected to enzyme-linked immunosorbent assay for detection of cytokine (IL-1 and IL-8) production levels. The experiment was repeated three times and the results are presented as the mean with standard deviation.

### 2.8. Data Analysis

All of the data were analyzed with SPSS 17.0 software (IBM, Armonk, NY, USA) according to the statistics. The difference was considered statistically significant if the *p*-value was less than 0.05.

## 3. Results

### 3.1. ERK1/2 Phosphorylation Increased throughout C. trachomatis Growth

**Figure 1:**
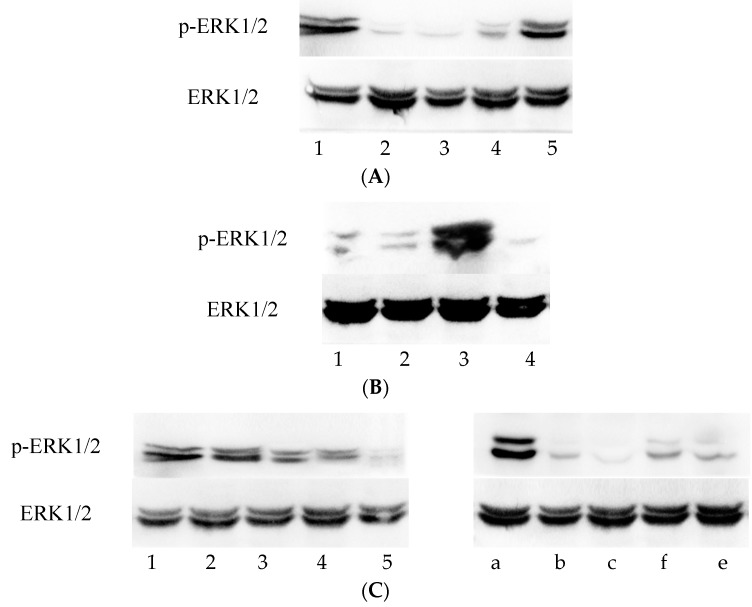
The amount of protein loaded in each lane is 100 μg. ERK1/2 was set as a loading control at the same time. ERK1/2, extracellular signal-regulated kinase 1 and 2; p-ERK1/2, phosphorylated of ERK1/2. (**A**) The Western blotting of p-ERK1/2 and ERK1/2 for Group C (the *Chlamydia trachomatis* group) at different time points. Lanes 1 to Lane 5 represent the time points of 0, 12 h, 24 h, 36 h, and 48 h, respectively; (**B**) The Western blotting of p-ERK1/2 and ERK1/2 for four groups after 48 h of incubation. 1: Group C + A, the azithromycin-treated group. 2: Group C + V, the Vp1-treated group. 3: Group C, the *Chlamydia trachomatis* group. 4: Group M, the blank group (only McCoy cells); (**C**) The Western blotting of p-ERK1/2 and ERK1/2 for Group C + V and Group C + A during the incubation at different time points. Lanes 1 to Lane 5 represent the time points of 0, 12 h, 24 h, 36 h, and 48 h for Group C + V, respectively. Lanes a–e represent the time points of 0, 12 h, 24 h, 36 h, and 48 h for Group C + A, respectively.

**Figure 2:**
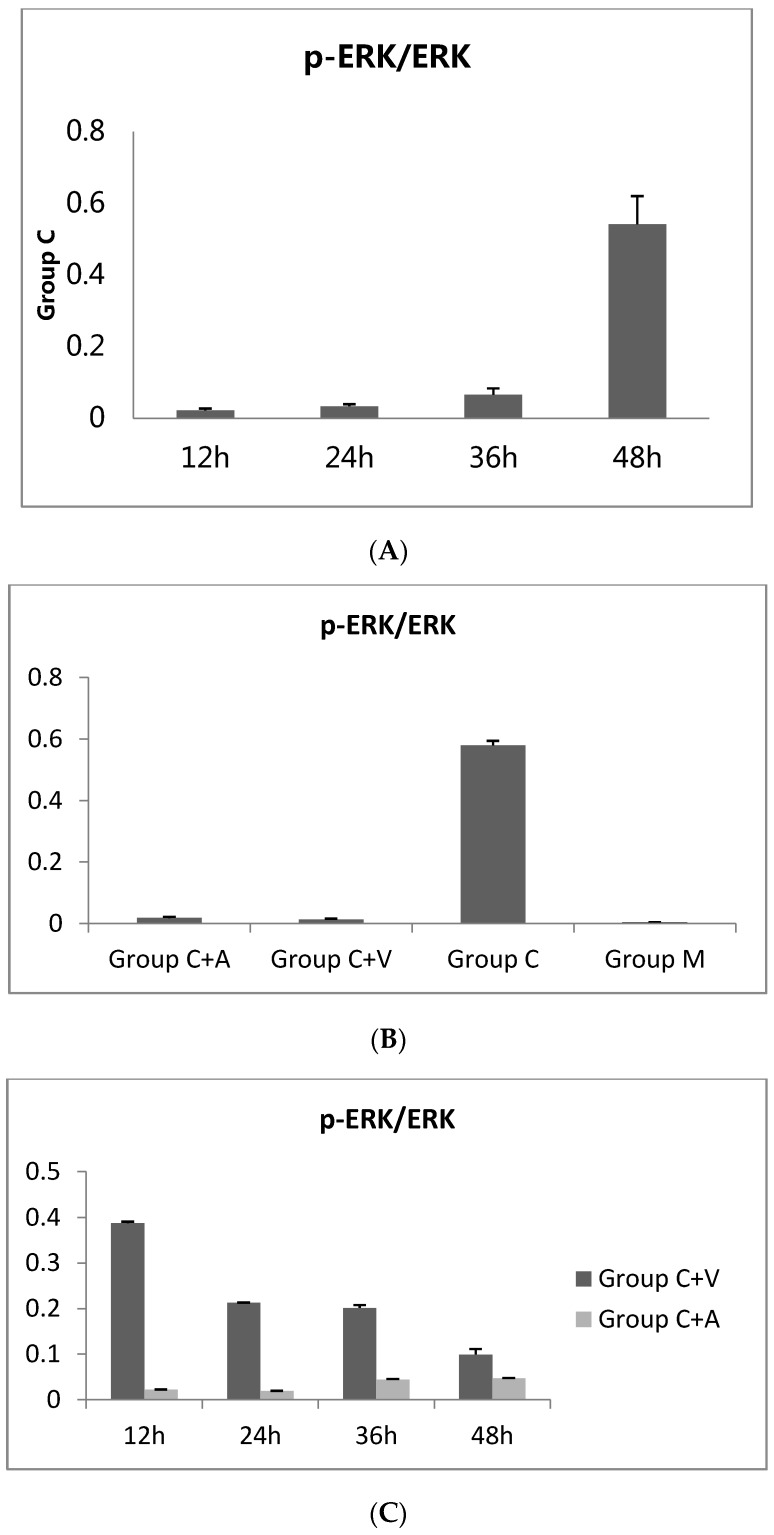
Grey value ratios of the ratio of p-ERK1/2 to ERK1/2 expression (**A**). Group C at different time points. * Statistically significant difference between the value at 48 h and other three other time points by one-way ANOVA, LSD (Least Significant Difference) (*p* = 0.000); (**B**) four groups after 48 h of incubation. * Statistically significant difference compared with Group C by the Paris *t*-test (*p* = 0.000); (**C**) Group C + V and Group C + A at different time points during the incubation. * Statistically significant difference compared with other three time points by one-way ANOVA, LSD (*p* = 0.000). ** Statistically significant difference compared with the value of 12 h (*p* = 0.000). # Statistically significant difference compared with the value of 12 h (*p* = 0.028) and 24 h (*p* = 0.017). ## Statistically significant difference compared with the value of 12 h (*p* = 0.016) and 24 h (*p* = 0.009). Group C + A, the azithromycin-treated group. Group C + V, the Vp1-treated group. Group C, the *Chlamydia trachomatis* group. Group M, the blank group (only McCoy cells).

During the culture of cells in Group C, which only contained the *Chlamydia*, the expression of p-ERK1/2 protein showed a tendency to gradually increase (Figure 1A). The p-ERK1/2 levels substantially decreased from 0 to 12 h, as determined by the decrease in the grey value ratio from 0.662 ± 0.039 to 0.022 ± 0.005. This initial decrease was likely due to the presence of 1 mg/mL cycloheximide in the culture medium, which is an antibody against actinomycetes and inhibits protein synthesis. However, in the subsequent period of *Chlamydia* culture, 12–48 h, the expression of p-ERK1/2 gradually increased again, accompanied with an increase in the grey value ratio from 0.022 ± 0.005 to 0.542 ± 0.078. This result indicates that the *Chlamydia* activates ERK1/2 phosphorylation, confirming that *C. trachomatis* growth relies on the MEK/ERK pathway. The change in p-ERK1/2 levels over time was statistically significant (Figure 2A).

### 3.2. p-ERK1/2 Expression Was Reduced in the Vp1- and Azithromycin-Treated Cells as the Infection Rate Decreased

**Figure 3:**
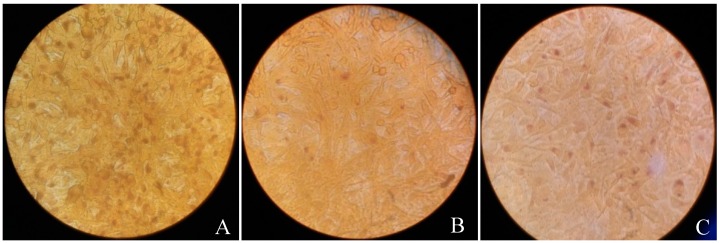
The inclusions viewed under the microscope (×200) by iodine dye. The inclusions are stained dark brown and positioned decentered in the cells, whereas the normal cells are light-stained or uncolored. (**A**) the inclusions of *Chlamydia trachomatis* infection after 48 h; (**B**) the inclusions of Vp1-treated *Chlamydia trachomatis* infection after 48 h; (**C**) the inclusions of azithromycin-treated *Chlamydia trachomatis* infection after 48 h.

The number of inclusions decreased after 48 h of incubation in both Group C + V and Group C + A (Vp1-treated and azithromycin-treated infected cells, respectively) compared to that of Group C (*C. trachomatis* infection-only). The infection rate of Group C was over 90%, with 63503.40 ± 2216.13 inclusions per well. The inhibition rate of Group C + V and Group C + A reached up to 76.48% (14933.00 ± 1814.88 inclusions) and 64.45% (22578.26 ± 2583.70 inclusions), respectively (Figure 3).

Furthermore, significant differences in p-ERK1/2 expression levels were observed between the *Chlamydia*-infected groups (C + V, C + A, and C) and the untreated control group (M) after 48 h of incubation (Figure 1B). The p-ERK1/2 expression level of Group C (0.580 ± 0.014) was much higher than that of Group M (0.004 ± 0.000), whereas those of Group C + V (0.013 ± 0.002) and Group C + A (0.018 ± 0.004) were lower than that of Group C (Figure 2B). This result indicates that, similar to azithromycin, the inhibition effect of Vp1 occurs through the MEK/ERK pathway.

### 3.3. Vp1 Inhibits C. trachomatis in the Late Infection Period, Whereas Azithromycin Inhibits Growth in the Early Period

The results described above clarified that Vp1 and azithromycin definitely inhibit the growth of *Chlamydia trachomatis*. The p-ERK1/2 levels of the cells were lower than the pre-intervention levels. Considering the changes over time in greater detail, in Group C + V, the p-ERK1/2 level gradually decreased from 12 h to 48 h, whereas the decrease in Group C + A only started at 12 h to 24 h and was maintained at a low level until 48 h (Figure 1C and Figure 2C). This result indicated that Vp1 exerts its effect on *Chlamydia* in the late infection period (36–48 h), whereas the inhibitory effect of azithromycin occurs in the early infection period (12–24 h).

### 3.4. The ERK1 Gene Was Up-Regulated and the ERK2 Gene Was Downregulated after C. trachomatis Infection

After 48 h of incubation, the relative *ERK1* mRNA expression levels of cells in Groups C, C + V, and C + A were all increased compared with that of Group M. The expression level of the *Chlamydia* infection group showed a 2.380-fold increase compared to the uninfected control cells. Furthermore, the *ERK1* mRNA levels of the Vp1-treated and azithromycin-treated group showed a 1.659- and 1.915-fold increase compared to the control level, respectively. By contrast, the relative *ERK2* mRNA expression levels of all three groups decreased after 48 h of incubation compared with the control group. The expression level of the *Chlamydia* group decreased by 0.883-fold compared to the control, whereas the Vp1- and azithromycin-treated groups showed larger decreases of 0.827- and 0.797-fold compared to the control.

### 3.5. IL-8 and IL-1 Levels Decreased after Vp1 and Azithromycin Treatments

**Figure 4:**
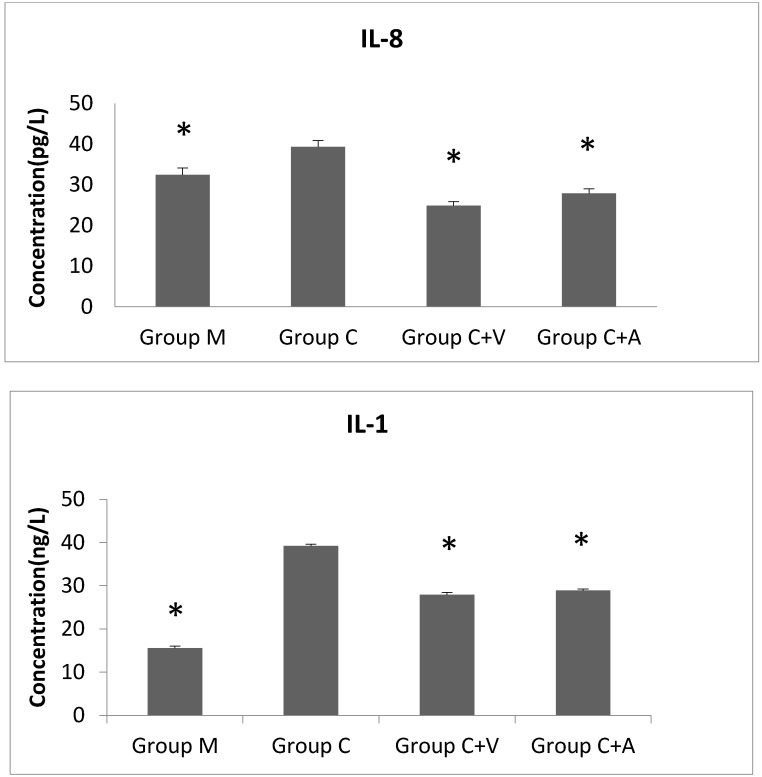
IL-8 and IL-1 production for four groups after 48 h of incubation. The experiment was repeated three times and the results are shown as mean with standard deviation. * Statistically significant difference compared with Group C by the Paris *t*-test (*p* = 0.000). Group C + A, the azithromycin-treated group. Group C + V, the Vp1-treated group. Group C, the *Chlamydia trachomatis* group. Group M, the blank group (only McCoy cells).

The mean IL-8 production level increased when the cells were infected with *Chlamydia* compared to the uninfected control group (Group M) after 48 h, whereas the IL-8 levels sharply decreased in the Vp1- and azithromycin-treated groups (Figure 4). From 0 to 48 h of incubation, the IL-8 levels of Group M were relatively stable, with a slightly early decrease and subsequent increase. However, Group C showed a considerable reduction in IL-8 production at 0–24 h and rapid growth from 24–48 h. The Vp1-treated group showed a sharp increase at 0–12 h and a gradual decrease at 12–48 h. The IL-8 level of the azithromycin-treated group increased at 0–24 h, and decreased at 24–48 h. Similar tendencies over time and between groups were observed for IL-1 production. The IL-1 level of Group C was increased comparing to that of the control, Group M. The IL-1 levels of the Vp1-and azithromycin-treated groups were reduced compared to those of Group C (Figure 4). During the incubation, IL-1 levels of Group M decreased from 0–12 h and were relatively stable from 12 h to 48 h. Group C showed an increasing tendency from 12 h to 48 h. The IL-1 level of the Vp1-treated group decreased from 0 to 24 h, increased from 24 h to 36 h, and further decreased from 36 h to 48 h. Although the azithromycin-treated group showed a similar tendency to the Vp1-treated group, its peak was observed at 24 h.

## 4. Discussion

*C. trachomatis* urogenital tract infection is a sexually transmitted disease that has become increasingly common and is a global health concern. The mechanisms of *Chlamydia* infection are closely related to inflammation-induced pathologic damage. Accordingly, the MAPK signaling pathways play a major role in the *C. trachomatis* infection, especially with respect to the growth and production of pro-inflammatory cytokines. To further understand the mechanisms of *Chlamydia* infection, it is important to investigate the mechanisms underlying the inhibition of *C. trachomatis* by the bacteriophage capsid protein Vp1. We here report the first attempt at exploring the relationship between *C. trachomatis*-infected cells treated with Vp1 and the MEK/ERK pathway at the protein and gene levels. Furthermore, we evaluated the effects of Vp1 treatment on the pro-inflammatory cytokines IL-8 and IL-1 to clarify the mechanism of inhibition.

First, we found that the p-ERK level increased gradually during *C. trachomatis* incubation with McCoy cells, which confirms Du *et al*. [8] in HeLa cells. In addition, Vp1 decreased the p-ERK level and the *ERK1* and *ERK2* mRNA expression levels, showing the same effects as azithromycin treatment. These results demonstrate that Vp1 and azithromycin have the same mechanism of *Chlamydia* inhibition by modulating the MEK/ERK pathway and minimizing ERK activation. The only difference was the effective time of the inhibition: the Vp1 is effective in the late infection period, while azithromycin was effective in the early period of *Chlamydia* growth.

Second, the *ERK1* mRNA level was upregulated, while the *ERK2* mRNA level was downregulated after *C. trachomatis* infection. Although the *ERK1* and *ERK2* genes have 90% sequence identity and share the same target upstream and downstream, they have some different biological functions. For example, ERK2 plays a key role in hepatocyte cell division, while ERK1 improves long-term hepatocyte survival [18]. In addition, specifically ERK2 but not ERK1 is required for the c-Met-paxillin signaling axis in HGF (Hepatocyte Growth Factor)-mediated motility [14]. The results of this study suggest that ERK1 might play a more important role in *C. trachomatis* growth than ERK2. However, confirmation of this hypothesis and the detailed mechanism await further investigation.

Finally, the pro-inflammatory cytokines IL-8 and IL-1 were found to increase after *C. trachomatis* infection, confirming previous research [11]. These two cytokines are directly induced by ERK pathways. In our study, production of these two cytokines also decreased after infected cells were treated with Vp1 and azithromycin compared with the untreated group. The IL-8 level of the Vp1-treated group decreased earlier than that of the azithromycin-treated group, while the IL-1 of the both groups showed an initial decrease (similar to the untreated infected cells), and then peaked at 24 h and 36 h before decreasing subsequently. The observed variations of IL-8 and IL-1 provide further understanding of the function of related pathways and their relationship to *Chlamydia* inhibition.

## 5. Conclusions

In conclusion, our research demonstrates that the mechanism of chlamydiaphage φCPG1 capsid protein Vp1 inhibiting *C. trachomatis* growth is associated with the MAPK pathway, via inhibition of the pro-inflammatory cytokines IL-8 and IL-1. Therefore, we have identified that bacteriophages may also influence their hosts via signaling transduction mediation and not only via bacteriolysis. Thus, we have uncovered a likely preliminary mechanism for how Vp1 inhibits *C. trachomatis* in cells. Nevertheless, the specific mechanisms determining the relationship between Vp1 and *C. trachomatis* are still under exploration.

Although azithromycin is still the first-choice recommended treatment for *C. trachomatis* [19], its clinical use has become less frequent because of acquired drug resistance. Although a chlamydia vaccine has been under exploration for several years and several candidate antigens have been screened, vaccine development remains a great challenge [20]. Therefore, the exploration of infection mechanisms offers new perspective for *Chlamydia* treatment. Bacteriophages have unique characteristics and show good potential as a clinical treatment to overcome antibiotic resistance [21]. The use of bacteriophages would minimize the chance of secondary infections because of their specific combination with hosts, which is a continued risk with antibiotics. In addition to their high specificity, the concentration at the site of infection can be assured so that side effects can be limited. Experts make efforts on phage transformation to get broad-spectrum, long-life and multi-function phages [22]. Therefore, the application of chlamydiaphages as a biotic therapy shows good future promise and warrants further exploration.

## Figures and Tables

**Table 1:** Design groups for treatment.

	Group C	Group C + V	Group C + A	Group V	Group A	Group M
*C. trachomatis*	√	√	√			
Vp1		√		√		
Azithromycin			√		√	
McCoy cell	√	√	√	√	√	√

Notes: C: *C. trachomatis*, V: Vp1, A: Azithromycin, M: McCoy cell.

**Table 2:** The primer sequences used for PCR.

Gene	Primer Sequences (5′ to 3′)	Products Size (bp)
Mapk1 (ERK2)	F GTATTCTTGGATCTCCATCACAGG	246
	R TGGGCTCATCACTTGGGTCA	
Mapk3 (ERK1)	F CAAACAAGCGCATCACAGTAGA	113
	R CAGCTCCATGTCGAAGGTGAAT	
Actin	F GCCTTCCTTCTTGGGTAT	97
	R GGCATAGAGGTCTTTACGG	
